# TNF-*α *and IL-10 downregulation and marked oxidative stress in Neuromyelitis Optica

**DOI:** 10.1186/1476-9255-6-18

**Published:** 2009-06-02

**Authors:** Giselle Pentón-Rol, Majel Cervantes-Llanos, Gregorio Martínez-Sánchez, José A Cabrera-Gómez, Carmen M Valenzuela-Silva, Omar Ramírez-Nuñez, Mayté Casanova-Orta, María A Robinson-Agramonte, Ileana Lopategui-Cabezas, Pedro A López-Saura

**Affiliations:** 1 Clinical Trials Division, Center for Biological Research, Havana, Cuba; 2 Center for Research and Biological Evaluations, Institute of Pharmacy and Food Sciences, University of Havana, Havana, Cuba; 3 International Center of Neurological Restoration, Havana, Cuba; 4 Higher Institute of Medical Sciences "Victoria de Girón", Havana, Cuba

## Abstract

**Background:**

Neuromyelitis optica is a central nervous system demyelinating and inflammatory syndrome. The objective of this study is to identify cytokines related to the cellular immune response as well as blood brain barrier integrity and oxidative stress.

**Methods:**

We performed a molecular characterization of cellular immune response and oxidative stress in serum from relapsing-NMO (R-NMO) patients and established the correlations between the clinical measurements and molecular parameters using the Bayesian approach.

Serum samples from 11 patients with R-NMO diagnosed according to Wingerchuk criteria and matched in terms of age, gender and ethnicity with the healthy controls were analyzed. The levels of TNF-*α*, IFN-*γ*, IL-10, MMP-9, TIMP-1 and oxidative stress markers: malondialdehyde, advanced oxidation protein products, peroxidation potential, superoxide dismutase, catalase, and total hydroperoxides were measured.

**Results:**

We found almost undetectable levels of TNF-*α*, a decreased production of IL-10 and a significant up-regulation of every oxidative stress biomarker studied. The insufficient production of TNF-*α *and IL-10 in R-NMO patients, which are two important players of T cell mediated immunoregulation, suggest an effector – regulator imbalance. The overproduction of oxygen reactive species as a consequence of the chronic inflammatory milieu is reflected on the excess of oxidative damage mediators detected. Furthermore, Multidimensional Scaling and a Bayesian linear regression model revealed a significant linear dependence between Expanded Disability Status Scale Kurtzke and TIMP-1; pointing to a possible predictive or prognostic value of this clinical-molecular relationship.

**Conclusion:**

These results suggest that there is a breakdown in immunoregulatory mechanisms and noteworthy pro-oxidant environment contributing to NMO pathogenesis.

## Background

NMO is a neurological disease clinically characterized by severe optic neuritis and transverse myelitis, where the clinical monophasic and relapsing forms are distinguished. Recently recognized as a distinct clinical entity from Multiple Sclerosis (MS)[1], NMO patients show B cell autoimmunity; while NMO-IgG, an antibody that targets aquaporin-4, was found in the serum of 70% of the patients [2].

Plasmapheresis and immunosuppressive therapies are beneficial but they do not eliminate the disease, thereby suggesting other pathogenic mechanisms such as cellular immune response and oxidative stress (OS).

Since immunoregulation highly contributes to homeostasis, its loss, and the consequent rupture of the balance between the effector and regulator environments, could be one of the etio-pathogenic mechanisms of NMO.

Oxidative stress is defined as an imbalance in the generation of reactive oxygen species (ROS) and plays a major role in disease pathogenesis. The immune release of ROS has been involved in demyelination and axonal damage; while the weak cellular antioxidant defense systems in the CNS and its vulnerability to ROS may increase damage[3].

Here we study the relevance of the cellular immune response and OS in NMO. Serum levels of TNF-*α*, IFN-*γ*, IL-10, MMP-9 metalloproteinase, its inhibitor TIMP-1 and OS markers: malondialdehyde, advanced oxidation protein products, peroxidation potential, superoxide dismutase, catalase, and total hydroperoxides from R-NMO patients and healthy controls (HC) were measured. We also explored the correlation between clinical measurements and molecular parameters for associations that may predict or act as prognostic factors of NMO.

## Methods

### Patients

Serum samples from 11 patients with R-NMO diagnosed according to Wingerchuk et al. [4] were analyzed in the present study. Eligibility criteria included no evidence of relapse and no corticosteroids, immunomodulatory or immunosuppressive treatment for at least 30 days previous sample collection.

Serum samples from apparently healthy donors were used as controls. HC were matched in terms of age, gender and ethnicity with the group of patients enrolled in the study. After collection, specimens were stored at -70°C until use. Written informed consent of each subject was obtained prior to sample collection. The study protocol was approved by the institutional review board according to the guidelines of the national legislation and the Code of Ethical Principles for Medical Research Involving Human Subjects of the World Medical Association.

### ELISA

Quantification of serum levels of cytokines, chemokine, matrix metalloproteinase and its inhibitor were assessed using commercially available quantitative sandwich ELISA kits according to instructions of the manufacturers. Cytokines included hu-IFN-*γ *(High-Sensitivity Assay, Catalog Number RPN2787, Biotrak, GE Healthcare UK Ltd Amersham Place, England), hu-TNF-*α *(Quantikine^® ^HS High Sensitivity, Catalog Number HSTA00C, R&D Systems, Minneapolis, MN), hu-IL-10 (Quantikine^® ^HS High Sensitivity, Catalog Number HS100B, R&D Systems, Minneapolis, MN), hu-CCL2 (Quantikine^®^, Catalog Number DCP00, R&D Systems, Minneapolis, MN), hu-MMP-9 (total) (Quantikine^®^, Catalog Number DMP900, R&D Systems, Minneapolis, MN) and hu-TIMP-1 (Quantikine^®^, Catalog Number DTM100, R&D Systems, Minneapolis, MN).

In brief, microtiter strips pre-coated with monoclonal antibodies generated against the proteins were used for quantification. After adding standards or samples and washing away any unbound molecule, a second enzyme- or biotin-conjugated antibody was added to the wells. Following washes, a chromogenic substrate for the enzyme or streptavidin-peroxidase and then a chromogenic substrate for biotin were added to the wells. A stopping solution was added and the intensity of the color was measured. The standard curve was used to extrapolate sample protein levels.

All samples were analyzed undiluted and duplicated.

### Biochemical determinations

All biochemical parameters were measured with spectrophotometric methods. The biochemical markers determined are: advanced oxidation protein products (AOPP); Malonyldialdehyde (MDA); peroxidation potential (PP); superoxide dismutase (SOD); catalase (CAT); total hydroperoxides (THP).

### Advanced Oxidation Protein Products

The AOPP were measured through the oxidation of iodide anion to diatomic iodine by AOPP [5]. and were calibrated with chloramine-T solutions as described elsewhere [6]. AOPP concentrations were expressed as micromoles per liter of chloramine-T equivalents.

### Malondialdehyde

Concentrations of MDA were analyzed using the LPO-586 kit obtained from Calbiochem (La Jolla, CA). In the assay, the production of a stable chromophore after 40 min of incubation at 45°C was measured at 586 nm. Freshly prepared solutions of malondialdehyde bis [-dimethyl acetal] (Sigma) was used and assayed as the standard under identical conditions [7].

### Peroxidation Potential

The PP was measured inducing lipid peroxidation by adding Cu^+ ^(2 mM) to the serum (incubated for 24 h at 37°C), to determine the balance between pro-oxidant-antioxidant factors. The difference between MDA concentrations, measured at 0 and 24 h after induction, was calculated for each sample[8].

### Superoxide Dismutase

Total SOD activity was measured using pyrogallol as the substrate[9]. This method follows the superoxide driven auto-oxidation of pyrogallol at pH 8.2 in the presence of ethylenediaminetetraacetic acid (EDTA). The standard assay mixture contained 1 mM EDTA in 50 mM Tris(hydroxymethyl)-aminomethan-HCl buffer (pH 8.2) with or without the sample. The reaction was started by the addition of pyrogallol (final concentration 0.124 mM) and the oxidation of pyrogallol was followed for 1 min at 420 nm. The percentage of inhibition of the auto-oxidation of pyrogallol by the SOD present in the serum sample was determined, and standard curves were established using known amounts of purified SOD (Sigma) under identical conditions. One unit of SOD activity was defined as the amount that reduced the absorbance change by 50%, and results were expressed as U·mL·^-1^min·^-1^.

### Catalase

CAT activity was measured by following the decomposition of hydrogen peroxide at 240 nm at 10 second intervals for one min[10].

### Total Hydroperoxides

Quantification of THP was measured by Bioxytech H_2_O_2_-560 Cat. 21024 kit, (Oxis internacional Inc. Portland, USA). The assay is based on the oxidation of ferrous ions to ferric ions by hydroperoxides under acidic conditions. The ferric ions bind with the indicator dye xylenol orange (3,3'-bis(N,N-di(carboxymethyl)-aminomethyl)-o-cresolsulfone-phatein, sodium salt) to form a stable coloured complex, which can be measured at 560 nm.

### Statistical analysis

To analyze immunoregulatory and oxidative stress molecular markers we estimated the mean difference between patients and the controls and tested the hypothesis H_0_: D ∈ [d1, d2] vs. H_1_: D<d1 ó D>d2 (D: difference of the means) with a Bayesian approach, estimating the Bayes factor and the probability that H_0 _is true. To detect similarities or dissimilarities (distances) between immunoregulatory and oxidative stress molecular markers with respect to clinical stage (EDSS) and number of relapses, we used the Multidimensional Scaling as a multivariate exploratory technique and then we considered a bayesian linear regression on the expectation of "y = EDSS" assuming "non-informative" distributions *a priori *for the parameters of the model.

## Results

Serum levels of Th1 (TNF-*α*, IFN-*γ*)/Th2-regulatory (IL-10) cytokines were evaluated in R-NMO patients and HC.

**Figure 1:**
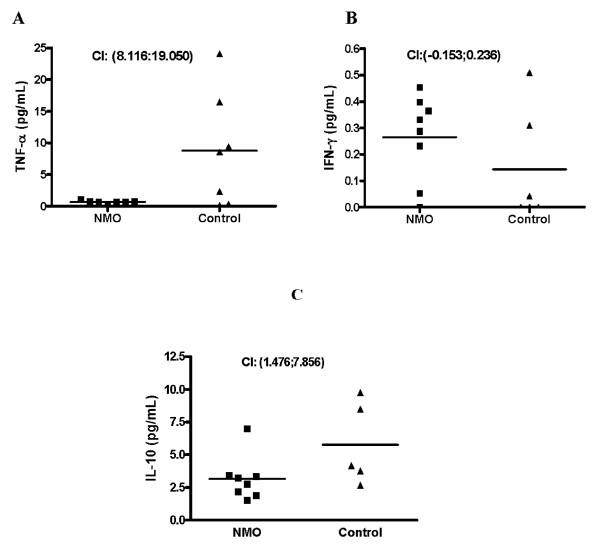
**Serum levels of cytokines in NMO patients and HC measured by ELISA**. **(A) **TNF-*α*; **(B) **IFN-*γ *and **(C) **IL-10. **CI**: Confidence interval for the difference "HC-NMO".

NMO patients had a TNF-*α *level lower than HC (Figure 1A). The confidence interval for the difference (CI 95%) "HC-NMO" was (CI: 8.116; 19.050), indicating a difference greater than 8 units in HC with a high probability (0.976). Interestingly, the levels of this cytokine were uniformly close to zero in all NMO patients. No significant differences were observed in IFN-*γ *(Figure 1B) levels and the measurements resulted widely dispersed in both groups.

Concerning IL-10, we obtained a significant down-regulation in R-NMO patients compared to controls. (CI: 1.476; 7.856), indicating a difference greater than 1.5 units in HC with a high probability (0.956) (Figure 1C).

**Figure 2:**
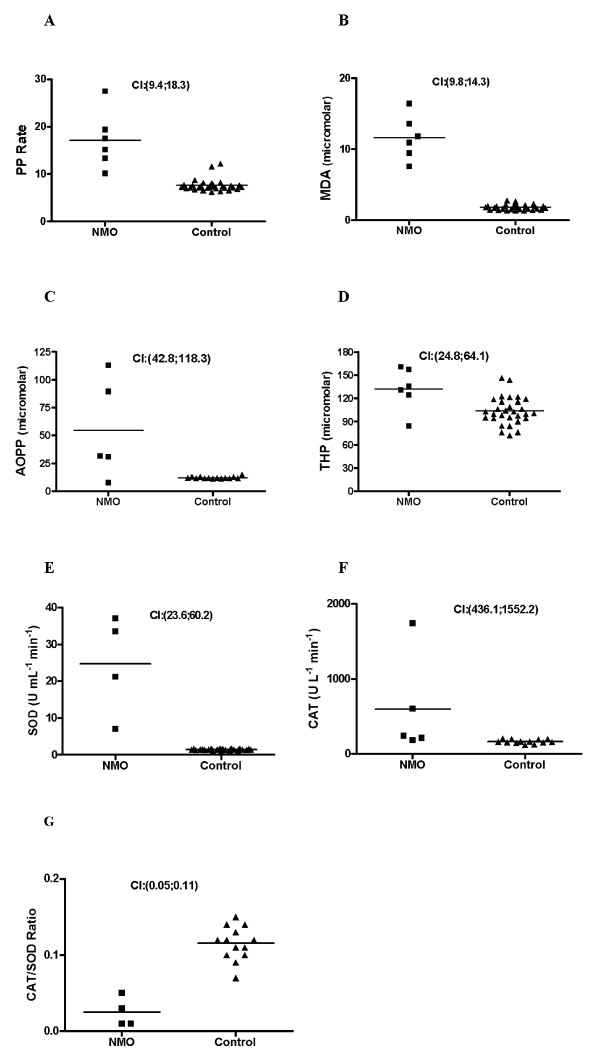
**Serum levels of oxidative stress markers**. **(A) **PP; **(B) **MDA; **(C) **AOPP; **(D) **THP; **(E) **SOD; **(F) **CAT and **(G) **CAT/SOD ratio. **CI**: Confidence interval for the difference "HC-NMO".

Lipid peroxidation, which may cause oligodendrocytic damage, was assessed through MDA concentration and PP evaluation. In fact, our results indicated a significant up-regulation of the PP with a probability for the difference to be over 0 of 0.990 (Figure 2A) and therefore of the MDA with a probability for the difference to be over 0 of 0.986 (Figure 2B) in R-NMO patients compared to HC.

Proteins are major targets for radicals and other oxidants when these are formed in both intra- and extracellular environments in vivo. Damaged proteins may be highly sensitive protein-based biomarkers of oxidative processes in mammalian systems. Oxidized proteins are often functionally inactive and their unfolding is associated with enhanced susceptibility to proteinases. Here, we found that the AOPP and THP were also up-regulated in R-NMO patients (the probability that the difference be over 0 is 0.970 for AOPP and 0.976 for THP) (Figures 2C and 2D).

In our results we found that SOD and CAT enzymes were up-regulated in R-NMO patients compared to HC (probability for the difference to be over 0 of 0.987 for SOD and 0.974 for CAT) suggesting the activation of a detoxification feedback mechanism in turn to abolish the excess of superoxide radicals as a result of the CNS inflammatory process (Figures 2E and 2F). However, CAT/SOD ratio was down-regulated in NMO patients compared to HC (Figure 2G).

We evaluated the serum levels of the metalloproteinase MMP-9 and its inhibitor TIMP-1 from R-NMO patients and HC (data not shown) and significant variations were not found.

**Figure 3:**
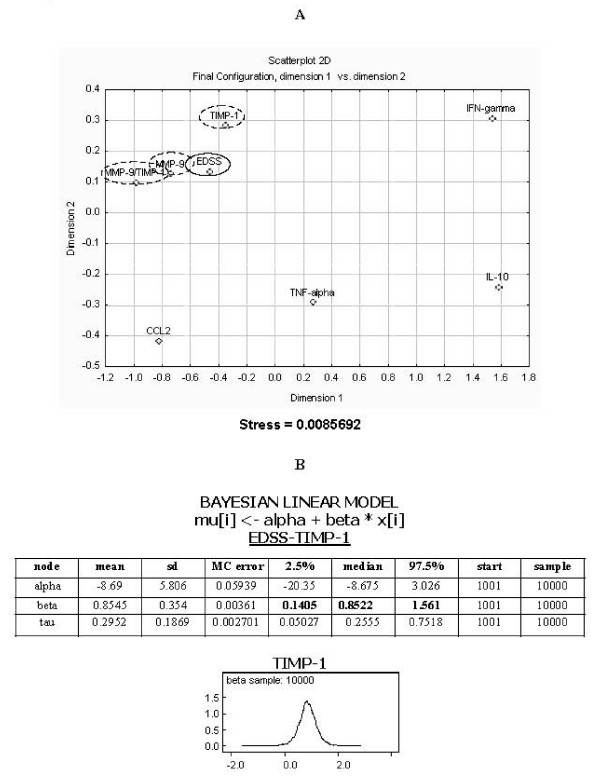
**Correlations between clinical and molecular parameters**. **(A) **Final configuration to Multidimensional Scaling (MDS). **(B) **Adjusted Bayesian linear model between EDSS and TIMP-1. Stress: Degree of correspondence between the distances among points implied by MDS map and the matrix of the observed data.

Furthermore, correlations between MMP-9, TIMP-1 and MMP-9/TIMP-1 and EDSS scale and number of relapses in NMO patients were determined. Figure 3A shows the final configuration to MDS viewing the distance (based on two dimensions) between EDSS and molecular parameters in patients. We can observe that EDSS is as close to TIMP-1 and MMP-9 as is the relation MMP-9/TIMP-1 with a high degree of correspondence between the distances among points implied by the MDS map and the matrix input, by a stress of 0.0086.

In order to establish the specific relation between these variables, we adjusted a Bayesian linear model:



where τ is the precision of the normal distribution *τ *= *σ*^-2^

with minimally informative distributions *a priori *for the parameters of the model:



There is a significant linear dependence between EDSS and TIMP-1 as can be observed in the graphic of density function (Figure 3B), the confidence interval does not contain 0, thereby an increase of the TIMP-1 values, represents an increase of the EDSS scale.

A similar analysis was done for the EDSS-oxidative stress markers and the number of relapses with all molecular parameters. We did not find any correlation between clinical and molecular markers (data not shown).

## Discussion

One theory of immune regulation involves homeostasis between Th1 and Th2 activity. Overactivation of either pattern can cause disease and either pathway can down-regulate the other. But the hypothesis has major inconsistencies, human cytokine activities rarely fall into exclusive pro-Th1 or -Th2 patterns. The regulatory T cells, likely influence immunity in a manner comparable to Th1 and Th2 cells. Many diseases previously classified as Th1 or Th2 dominant fail to meet the set criteria [11], therefore the most important element to considered is the effector (Th1 or Th2) – regulator (T regulatory cells) equilibrium which is lost in autoimmune diseases.

Some cytokines, such as TNF-*α*, IL-1 and IFN-*γ*, are well known for their promotion of inflammatory responses. However, these cytokines also have immunosuppressive functions and their subsequent expressions also assist in repair or recovery processes, suggesting a dual role for some pro-inflammatory cytokines[12].

TNF-*α *is considered an inflammatory cytokine that induces pleiotropic responses which comprise apoptosis in some cells and proliferation in others. Reports indicate either exacerbation or amelioration of pathological conditions in the brain during TNF-*α *treatment, including experimentally induced brain trauma [13] and a murine model of multiple sclerosis [14,15]. No clinical efficacy or worsening of the symptoms has been reported in some Multiple Sclerosis patients treated with TNF-*α *inhibitors [16]. In an animal model of demyelination/remyelination, the lack of TNF-*α *and TNFR2 provoked a significant reduction in oligodendrocytes leading to a delay in the generation of these cells, showing an unexpected role for TNF-*α *and TNFR2 in repair of the CNS [17]. In humans, beneficial effects of anti-TNF-*α *treatments in patients with autoimmune diseases are proved; though surprisingly a side effect of this treatment is the induction or exacerbation of humoral autoimmunity disorders[18]. Although controversial, human and murine studies suggest a link between reduced TNF-*α *production and the development of humoral autoimmunity, due to the prevention of CTL induction and the subsequent control of autoreactive B cells [19]. Recently, 15 cases of anti-TNF-*α*-associated optic neuropathy were reported [20], the effects of anti-TNF-*α *therapy may be related to changes in the balance of immunologic homeostasis [21].

According to our results, the TNF-*α *levels from NMO patients are practically undetectable evidencing that this cytokine is important to protect the CNS from the damage caused by the cascade of immune and inflammatory events that characterize this disease and consequently with reports that demonstrate neuroprotective [22,23] neurotrophic [24] and immunomodulatory actions of TNF-*α*.

Additionally, our results show a down-regulation of IL-10, a Th2 and regulatory cytokine, suggesting not a specific Th1/Th2 pattern but the imbalance of effector – regulator mechanisms is implied. Low serum levels of IL-10 have been reported in MS patients compared to HC, which have been found to be more drastic in patients with the progressive form of the disease [25] and treatments resulting in the up-regulation reduce the disease burden [26]. IL-10 levels of MS patients in remission were significantly higher than those in the active phase [27] indicating that in MS and probably in NMO, the regulatory cytokine, IL-10, is impaired.

Regulatory mechanisms are essential in preventing autoimmune disorders. Regulatory cytokines therapy is a tempting strategy for reestablishing the immune balance and thus preventing or reversing these disorders. Evidently, the down-regulation of TNF-*α *and IL-10 cytokines in NMO demonstrate a remarkable imbalance possibly responsible for the several events of the NMO pathogenesis. Our results are consistent to those obtained by others for MS [27] suggesting that it is possible to control NMO restoring the regulatory cytokines balance.

The role of OS in the progressive demyelinating NMO disease has not been studied. It is seductive to speculate that free radical oxygen chemistry contributes to pathogenesis in this condition [28].

Such oxidative stress can damage the lipids, proteins and nucleic acids of cells and mitochondria, potentially causing cell death. Oligodendrocytes are more sensitive to OS [29] in vitro than are astrocytes and microglia, seemingly due to a diminished capacity for antioxidant defense and the presence of raised risk factors, including high iron content. OS might therefore result in vivo in selective oligodendrocyte death, and thereby demyelination. The ROS may also damage the myelin sheath, promoting its attack by macrophages [30].

Damage can occur directly by lipid peroxidation and indirectly by the activation of proteases and phospholipase A2. Evidence for the existence of OS within inflammatory demyelinating lesions includes the presence of both lipid and protein peroxides.

Protein oxidation and enhanced proteolytic degradation, therefore, have been suggested to cause a net increase in ROS scavenging capacity. However, certain oxidized proteins are poorly handled by cells, and together with possible alterations in the rate of production of oxidized proteins, may contribute to the observed accumulation and damaging actions of oxidized proteins during several pathologies [31] such as shown by our results in NMO.

High activity of SOD in NMO patients may respond to an over generation of O_2_^•-^. If excess O_2_^•- ^is present, it can react with norepinephrine, dopamine and serotonin [32], to initiate their oxidation, which then continues with the production of more ROS, quinones, etc. Damage to brain readily releases iron (and copper) ions in forms capable of catalyzing free radical reactions. Catalytic iron released by the brain damage can persist because CSF has little or no iron-binding capacity [33].

There is a high activity of SOD in patients without the proportional increment of CAT to establish an appropriate balance CAT/SOD; in addition, the apparently normal level of THP, probably indicate an iron overload. In this condition the over production of H_2_O_2 _can occur. Neuronal damage can involve direct effects of H_2_O_2 _in inactivating sensitive enzymes as well as its reaction with iron ions to form •OH [33].

Matrix metalloproteinases are a family of enzymes found in the extracellular matrix. The control of the secretion of these proteases as well as the balance between MMP secretion and the secretion of their natural inhibitors TIMPs, has an important relevance in the pathogenesis of demyelinating diseases [34]. MMP-9/TIMP-1 ratio may be viewed as a reliable marker and may be predictive of MRI activity in relapsing-remitting MS [35].

In a comparative study on the MMP-9 and TIMP-1 levels in CSF from MS and NMO patients reported that the levels of MMP-9 and TIMP-1 in NMO were similar to HC resembling our results [36]. We also found that there is a specific relationship between MMP-9, TIMP-1 and MMP-9/TIMP-1 molecular parameters and EDSS clinical scale in a MDS map and a significant linear dependence between EDSS and TIMP-1. This result may suggest the relevance of these molecular parameters as a prognostic factor of the clinical measurement in the same way that it could have a predictive value for the MRI activity.

## Conclusion

Well designed clinical studies using pharmaceutical products directed towards the restoration of immuno-regulatory mechanisms, combined with an antioxidant or iron-chelating agents are needed to assess whether they could be beneficial for NMO treatment.

## Competing interests

The authors declare that they have no competing interests.

## Authors' contributions

GP-R, MC-Ll and GM-S participated in the conception and design of experiments, acquisition and analysis of data, interpretation of results and drafting the manuscript; IL-C performed the ELISA assays. OR-N and MO-G carried out the oxidative stress biochemical assays; JAC-G identified and classified the NMO patients; MAR-A participated in the NMO-IgG determinations; CV-S performed statistical analysis. PAL-S contributed in the drafting of the manuscript and revising it critically.

## References

[B1] Argyriou AA, Makris N (2008). Neuromyelitis optica: a distinct demyelinating disease of the central nervous system. Acta Neurol Scand.

[B2] Lennon VA, Wingerchuk DM, Kryzer TJ, Pittock SJ, Lucchinetti CF, Fujihara K, Nakashima I, Weinshenker BG (2004). A serum autoantibody marker of neuromyelitis optica: distinction from multiple sclerosis. Lancet.

[B3] Gilgun-Sherki Y, Melamed E, Offen D (2004). The role of oxidative stress in the pathogenesis of multiple sclerosis: the need for effective antioxidant therapy. J Neurol.

[B4] Wingerchuk DM, Lennon VA, Pittock SJ, Lucchinetti CF, Weinshenker BG (2006). Revised diagnostic criteria for neuromyelitis optica. Neurology.

[B5] Witko-Sarsat V, Friedlander M, Nguyen Khoa T, Capeillère-Blandin C, Nguyen AT, Canteloup S, Dayer JM, Jungers P, Drüeke T, Descamps-Latscha B (1998). Advanced oxidation protein products as novel mediators of inflammation and monocyte activation in chronic renal failure. J Immunol.

[B6] Witko VA, Nguyen T, Descamps-Latscha B (1992). Microtiter plate assay for phagocyte-derived taurine-chloramines. J Clin Lab Anal.

[B7] Esterbauer H, Cheeseman KH (1990). Determination of aldehydic lipid peroxidation product: malonaldehyde and 4-hydroxynonenal. Method in Enzymology.

[B8] Özdemirler G, Mehmetçik G, Oztezcan S, Toker G, Sivas A, Uysal M (1995). Peroxidation potential and antioxidant activity of serum in patients with diabetes mellitus and myocard infarction. Horm Metab Res.

[B9] Shukla GS, Hussain T, Chandra SV (1987). Possible role of superoxide dismutase activity and lipid peroxide levels in cadmium neurotoxicity: in vivo and in vitro studies in growing rats. Life Sci.

[B10] BOEHRINGER MANNHEIM (1987). Biochemica Information. A revised biochemical reference source. Enzymes for routine.

[B11] Kidd P (2003). Th1/Th2 balance: the hypothesis, its limitations, and implications for health and disease. Altern Med Rev.

[B12] Correale J, Villa A (2004). The neuroprotective role of inflammation in nervous system injuries. J Neurol.

[B13] Shohami E, Ginis I, Hallenbeck JM (1999). Dual role of tumor necrosis factor *α *in brain injury. Cytokine Growth Factor Rev.

[B14] Liu J, Marino MW, Wong G, Grail D, Dunn A, Bettadapura J, Slavin AJ, Old L, Bernard CC (1998). TNF is a potent anti-inflammatory cytokine in autoimmune-mediated demyelination. Nat Med.

[B15] Kassiotis G, Kollias G (2001). Uncoupling the pro-inflammatory from the immunosuppressive properties of tumor necrosis factor (TNF) at the p55 TNF receptor level. Implications for pathogenesis and therapy of autoimmune demyelination. J Exp Med.

[B16] The Lenercept Group (1999). TNF neutralization in MS: results of a randomized, placebo-controlled multicenter study. Neurology.

[B17] Arnett HA, Mason J, Marino M, Suzuki K, Matsushima GK, Ting JP (2001). TNF alpha promotes proliferation of oligodendrocyte progenitors and remyelination. Nat Neurosci.

[B18] Charles PJ, Smeenk RJ, De Jong J, Feldmann M, Maini RN (2000). Assessment of antibodies to double-stranded DNA induced in rheumatoid arthritis patients following treatment with infliximab, a monoclonal antibody to tumor necrosis factor alpha: findings in open-label and randomized placebo-controlled trials. Arthritis Rheum.

[B19] Via CS, Shustov A, Rus V, Lang T, Nguyen P, Finkelman FD (2001). In vivo neutralization of TNF-alpha promotes humoral autoimmunity by preventing the induction of CTL. J Immunol.

[B20] Journal compilation (2007). Royal Australian and Zealand College of Ophthalmologists. Letters to the Editor.

[B21] Robinson WH, Genovese MC, Moreland LW (2001). Demyelinating and neurologic events reported in association with tumor necrosis factor alpha antagonism: by what mechanisms could tumor necrosis factor alpha antagonism improve rheumatoid arthritis but exacerbate multiple sclerosis?. Arthritis Rheum.

[B22] Turrin NP, Rivest S (2006). Tumor necrosis factor alpha but not interleukin 1 beta mediates neuroprotection in response to acute nitric oxide excitotoxicity. J Neurosci.

[B23] Lastres-Becker I, Cartmell T, Molina-Holgado F (2006). Endotoxin preconditioning protects neurones from in vitro ischemia: role of endogenous IL-1beta and TNF-alpha. J Neuroimmunol.

[B24] Pickering M, Cumiskey D, O'Connor JJ (2005). Actions of TNF-alpha on glutamatergic synaptic transmission in the central nervous system. Exp Physiol.

[B25] Salmaggi A, Dufour A, Eoli M, Corsini E, La Mantia L, Massa G, Nespolo A, Milanese C (1996). Low serum interleukin-10 levels in multiple sclerosis: further evidence for decreased systemic immunosuppression?. J Neurol.

[B26] Ozenci V, Kouwenhoven M, Huang YM, Xiao B, Kivisäkk P, Fredrikson S, Link H (1999). Multiple sclerosis: levels of interleukin-10-secreting blood mononuclear cells are low in untreated patients but augmented during interferon-beta-1b treatment. Scand J Immunol.

[B27] Perrella O, Sbreglia C, Perrella M, Spetrini G, Gorga F, Pezzella M, Perrella A, Atripaldi L, Carrieri P (2006). Interleukin-10 and tumor necrosis factor-alpha: model of immunomodulation in multiple sclerosis. Neurol Res.

[B28] Calabrese V, Lodi R, Tonon C (2005). Oxidative stress, mitochondrial dysfunction and cellular stress response in Friedreich's ataxia. J Neurol Sci.

[B29] Kanwar JR (2005). Anti-inflammatory immunotherapy for multiple sclerosis/experimental autoimmune encephalomyelitis (EAE) disease. Curr Med Chem.

[B30] Smith KJ, Kapoor R, Felts PA (1999). Demyelination: the role of reactive oxygen and nitrogen species. Brain Pathol.

[B31] Martínez-Sánchez G, Giuliani A, Pérez-Davison G (2005). Oxidized proteins and their contribution to redox homeostasis. Redox Report.

[B32] Wrona MZ, Dryhurst G (1998). Oxidation of serotonin by superoxide radical: implications to neurodegenerative brain disorders. Chem Res Toxicol.

[B33] Halliwell B (2006). Oxidative stress and neurodegeneration: where are we now?. J Neurochem.

[B34] Cross AK, Woodroofe MN (1999). Chemokine modulation of matrix metalloproteinase and TIMP production in adult rat brain microglia and a human microglial cell line in vitro. Glia.

[B35] Avolio C, Filippi M, Tortorella C, Rocca MA, Ruggieri M, Agosta F, Tomassini V, Pozzilli C, Stecchi S, Giaquinto P, Livrea P, Trojano M (2005). Serum MMP-9/TIMP-1 and MMP-2/TIMP-2 ratios in multiple sclerosis: relationships with different magnetic resonance imaging measures of disease activity during IFN-beta-1a treatment. Mult Scler.

[B36] Mandler RN, Dencoff JD, Midani F, Ford CC, Ahmed W, Rosenberg GA (2001). Matrix metalloproteinases and tissue inhibitors of metalloproteinases in cerebrospinal fluid differ in multiple sclerosis and Devic's neuromyelitis optica. Brain.

